# Lessons from *Dwarf8* on the Strengths and Weaknesses of Structured Association Mapping

**DOI:** 10.1371/journal.pgen.1003246

**Published:** 2013-02-21

**Authors:** Sara J. Larsson, Alexander E. Lipka, Edward S. Buckler

**Affiliations:** 1 Department of Plant Breeding and Genetics, Cornell University, Ithaca, New York, United States of America; 2 USDA–Agricultural Research Service, Robert W. Holley Center for Agriculture and Health, Ithaca, New York, United States of America; University of Chicago and Howard Hughes Medical Institute, United States of America

## Abstract

The strengths of association mapping lie in its resolution and allelic richness, but spurious associations arising from historical relationships and selection patterns need to be accounted for in statistical analyses. Here we reanalyze one of the first generation structured association mapping studies of the *Dwarf8* (*d8*) locus with flowering time in maize using the full range of new mapping populations, statistical approaches, and haplotype maps. Because this trait was highly correlated with population structure, we found that basic structured association methods overestimate phenotypic effects in the region, while mixed model approaches perform substantially better. Combined with analysis of the maize nested association mapping population (a multi-family crossing design), it is concluded that most, if not all, of the QTL effects at the general location of the *d8* locus are from rare extended haplotypes that include other linked QTLs and that *d8* is unlikely to be involved in controlling flowering time in maize. Previous independent studies have shown evidence for selection at the *d8* locus. Based on the evidence of population bottleneck, selection patterns, and haplotype structure observed in the region, we suggest that multiple traits may be strongly correlated with population structure and that selection on these traits has influenced segregation patterns in the region. Overall, this study provides insight into how modern association and linkage mapping, combined with haplotype analysis, can produce results that are more robust.

## Author Summary

Eleven years ago, association mapping was a cutting-edge tool used to identify regions of a genome associated with phenotypic variation. One of the first association studies performed in plants was reported in Thornsberry, et al. (2001). Since then, researchers continued to develop new and improved genotyping, phenotyping, and statistical methods to examine the relationship between genotype and phenotype. Reanalysis of the old data for the *d8* locus and flowering time, as well as new and improved data sets, gives us a unique opportunity to examine the strengths and weaknesses of association studies. These new analyses reveal that the results reported in 2001 significantly overestimated the association between genotype and phenotype, in particular the estimated effect size. The key issues with the Thornsberry et al. (2001) study were lack of control for population structure and relatedness between individuals, as well as a potential confounding between the phenotype and the population structure examined. The new analysis demonstrates a marginal association between *d8* and flowering time, and a minimal effect (if any).

## Introduction

Association mapping, which was developed as a necessity for large-scale human studies, is commonly used in conjunction with family (linkage) mapping in plant and animal genetic studies. The application of association mapping for plants was originally assessed in Thornsberry J.M. (2001) [1] with Buckler as senior author. It was concluded that association mapping offers higher resolution than linkage mapping due to quicker linkage disequilibrium (LD) decay, that structured association mapping is crucial for controlling false positives arising from population structure, and that *Dwarf8* (*d8*) (RefGen_v2 position: Chr. 1; 266,094,769–266,097,836 bp) is associated with flowering time. This initial study has been cited extensively, and has been the basis of several reanalyses of *d8*. New data and statistical tools give us the opportunity to reevaluate this locus. Results show that the *d8* associations reported by Thornsberry et al. (2001) are likely false positives (i.e., spurious associations), which resulted from insufficient correction of population structure. Indeed, the application of association mapping to animal and plant studies has been very successful, culminating in many important findings [2]–[10]. In this light, the Thornsberry et al (2001) study has attracted a lot of interest to the area and led to more studies and the development of techniques to control for population structure and familial relatedness. When the phenotype is strongly correlated with population structure (e.g., flowering time), it is often difficult to obtain statistically significant results when the models used include covariates accounting for population structure. This leads to uncertainty when determining which associated sites are causative. Thus, linkage mapping is a valuable complementary approach in these situations, and in maize, large-scale connected mapping populations issued from diverse founders have been developed [11], [12] in order to conduct joint linkage-association analyses [10], [13], [14].

A major issue with association studies is false positives. In particular, indirect associations that are not causal will not be eliminated by increasing the sample size or the number of markers [15]. The main sources of such false positives are linkage between causal and noncausal sites, more than one causal site, and epistasis. These indirect associations are not randomly distributed throughout the genome and are less common than false positives arising from population structure. This makes them more difficult to control for than false positives arising from population structure.

The identification of a statistically significant association between a genotypic marker and a trait is considered to be proof of linkage between the phenotype and a casual site. This assumption is true for random mating populations with fast LD decay [16]. However, it is important to consider that population structure is typically present in association panels and it has an impact on the results. Population structure exists among all species in forms such as colonies, ethnic groups, and other subdivisions based on selection or geography. Typically, population structure leads to spurious associations between markers and the trait [17].

The ability to account for population structure in a given data set is influenced by the population size, the number of markers, the level of admixture, and the divergence in allele frequency between the subpopulations [18]. One commonly used method for controlling population structure is structured association (SA), which relies on randomly selected markers from the genome to estimate population structure. This estimate is then incorporated into the association analysis [16], [18], [19]. Another methodology for controlling population structure is to conduct a principal component analysis (PCA) [20], [21]. This approach summarizes the variation observed across all markers into a smaller number of underlying component variables. One interpretation of these principal components relates them to separate, unobserved subpopulations from which the individuals in the data set originate. The loadings (i.e., coefficient values) of the individuals for each principal component describe their relationship to the subpopulations. Both SA and PCA are limited to correcting for spurious associations by clustering on a global level of genetic variation. Thereby, they do not adequately capture the relatedness between individuals.

Correcting for population structure is not sufficient to eliminate all false positives. Therefore, the unified mixed linear model (MLM; also called the Q+K model) [22] was developed to further reduce the false positive rate by controlling for both population structure and cryptic familial relatedness. This approach uses a mixed model framework that has traditionally been used by animal geneticists [23], [24]. Specifically, covariates accounting for population structure are included as fixed effects (Q), and the individuals in the association panel are included as random effects. A kinship matrix (K) is calculated to estimate the variance-covariance between the individuals. Typically, the covariates used in the unified MLM are either principal components of the markers or covariates from SA approaches (e.g., STRUCTURE [17]). The advantages of the MLM are that it crosses the boundary between family-based and population-based samples. However, not all associations that are eliminated will be false. If a polymorphism is perfectly correlated with population structure, it is not possible to differentiate between true and false positives.

The initial study by Thornsberry et al. (2001) identified nine polymorphisms within *d8* [25] that were associated with variation in flowering time in an association panel consisting of 92 diverse inbred lines. The most significant site was an 18 bp deletion (RefGen_v2 position: Chr. 1; 266,094,529 bp) in the promoter region. A 6 bp indel (RefGen_v2 position: Chr. 1; 266,095,483) was also identified. This allele is over-represented in Northern Flint lines and is located near a Src Homology 2-like domain, which is an important binding domain within this class of transcription factors. The initial association analysis was performed using logistic regression analyses, accounting for population structure. Population structure was estimated as a modification of SA using STRUCTURE software [18] with *k* = 3.

Using a general linear model (GLM) without population structure, Andersen et al. (2005) obtained similar results for six of the nine *d8* polymorphisms identified by Thornsberry et al. (2001). However, when including population structure in the model, (using STRUCTURE with both *k* = 2 and *k* = 3 subpopulations), it was found that the association results were overestimated. Each subpopulation was also analyzed separately, and a spurious association was still detectable [26].

Camus-Kulandaivelu et al. (2006) examined the association between *d8* and flowering time using a panel of 375 inbred lines (including the 92 from the initial study) as well as a panel consisting of 275 traditional landraces from American and European origins [27]. Population structure was estimated using STRUCTURE, and association analysis was performed using both GLM and logistic regression. Their analysis revealed that the 6 bp indel at 266,095,483 bp (identified in Thornsberry et al., 2001) was spuriously associated with flowering time when covariates accounting for population structure were not included. In contrast, no association between *d8* and flowering time was detected in the inbred panel when accounting for population structure. However, this spurious association was still detectable in the traditional landraces panel, including Andean material that has no relationship to the Northern Flint material.

The *d8* gene produces a signaling factor involved in the gibberellin pathway. Gibberellins are types of endogenous plant growth regulators [28]. Maize *d8* and wheat *Rht-B1/Rht-D1* have been shown to be orthologous of the *GAI* gene [25]. Mutants of *d8* have severe height phenotypes due to alterations of the DELLA domain. In maize, these are dominant, gain-of-function mutations, suggesting that *d8* is a negative regulator. Conversely, recessive mutants of the *GAI* gene in *Arabidopsis* result in loss-of-function, specifically in polypeptides truncated upstream of the SH2-like domain. As a consequence, the gene product does not function as a negative regulator, resulting in normal height phenotypes [1].

Two evolutionary processes have likely impacted the *d8* locus. First, the associated allele, specifically the 6 bp indel reported in Thornsberry et al. (2001), is related with Northern Flint maize. Maize originated from southern Mexico, where there are long growing seasons and high temperatures. As maize agriculture expanded from Mexico through the Southwestern United States to the Eastern United States (with its shorter growing season and lower temperatures), a severe bottleneck occurred in maize diversity, resulting in the Northern Flint subpopulation [29]. The bottleneck created extensive long range LD in this subpopulation. Northern Flints were substantially isolated from all other maize subpopulations [29] until the introduction of the Southern Dents in the 1600s [30].

Additionally, the *d8* locus is located only 347,057 bp from the *tb1* (*teosinte branched1*) locus (RefGen_v2 position: Chr. 1; 265,745,979–265,747,712 bp), which is one of the key genes involved in maize domestication [31]. The *tb1* locus lost much of its diversity during the domestication process [31], [32]. The original *d8* study [1] identified evidence of purifying selection with substantial diversity loss; however, there was little LD identified in the region between *d8* and *tb1*. Although unconfirmed, some Northern Flint allied germplasm (e.g. sweet corn, P39) have a morphology that looks like the undomesticated *tb1* phenotype. It is likely that the region around *d8* and *tb1* has been through a bottleneck with multiple selective sweeps, resulting in complex extended haplotypes.

Most of the loci controlling flowering time in maize have been identified through QTL studies. Of these, only *d8* and *vegetative to generative transition 1* (*vgt1*) have been confirmed with association and fine mapping [33]. Located on chromosome 8, *vgt1* is arguably the most important flowering time locus in maize. It contains an APETALA2-like gene, *ZmRap2.7*, which is controlled by an enhancer region about 70 kb upstream [33]. The association between *vgt1* and flowering time is supported by a study conducted in the maize nested association mapping (NAM) population, where a major QTL was identified in this region [11]. This study also detected an allelic series at this QTL, suggesting that more than one causative allele is present. One of these alleles is from northern germplasm and is in linkage with a MITE whose association with early flowering time was confirmed in the NAM population [11]. Although the lack of the *vgt1* early flowering allele did not completely explain the late flowering time, a SNP identified in the *ZmRap2.7* gene showed association with the late flowering effect [11].

An association study by Ducrocq et al. (2008) [34] reported *P*-values for *vgt1* association several magnitudes lower that those obtained by Salvi et al. (2007) [33]. Both studies accounted for population structure. Compared to Salvi et al. (2007) [33], Ducrocq et al. (2008) used a more genetically diverse and larger association panel, including a higher number of lines from Northern Flint and European germplasm [34]. In the case of *d8*, the association between the site and the trait becomes less significant, and even undetectable, when increasing the number of lines examined. This supports no association between the 6 bp indel in *d8* and flowering time in maize. Including *d8* in the model when performing association mapping for flowering time does not change the result for the SNPs in *vgt1* [34]. This indicates that there is no interaction between the two loci.

The purpose of this study was to reanalyze the work of Thornsberry et al. (2001) utilizing some of the latest association mapping methodologies and data sets. This study compared association results from various statistical approaches using a maize diversity panel and the NAM population [11], [12]. Single nucleotide polymorphisms (SNPs) and insertions/deletions (indels) from recent genotyping efforts (e.g., HapMap sequencing from Gore et al. 2009 [35] and Chia et al. 2012 [36]) were used to evaluate these various approaches and the *d8* association.

## Results

### Association Study

The results from the Thornsberry et al. (2001) study showed significant association at both the 18 bp deletion (266,094,829 bp) in the promoter region and the 6 bp indel (266,095,483 bp). Our reanalysis of the two sites using the Q model and a significantly larger association panel (consisting of 282 lines) resulted in less significant associations at both loci (Table 1). By increasing the number of lines we are able to obtain a larger sample size within each of the subpopulations and thus, more accurately estimate the underlying population structure (i.e., Q).

**Table 1:** Association between polymorphisms at the *d8* locus and variation in flowering time in the 92 and 282 association panel, and association between polymorphisms in the region between *d8* and *tb1* (*d8*/*tb1*) and variation in flowering time in the 282 line association panel.

	SNP	Population	Naïve Model^a^	K^b^	Q^c^	Q+K^d^
			p-value	p-value	p-value	p-value
d8	18 bp	282	0.0775	0.3996	0.8422	0.3676
		92	7.95×10^−4^**	0.1585	0.0021*	0.0847
	3 bp	282	2.32×10^−5^**	0.5759	0.0077*	0.8697
		92	0.1649	0.1411	0.3143	0.2403
	6 bp	282	9.70×10^−6^**	0.0142*	0.0018*	0.0127*
		92	2.51×10^−5^**	3.23×10^−4^**	0.0012*	0.0017*
d8/tb1	36	282	0.0017*	0.309	0.005*	0.062
	72	282	0.003*	0.3123	0.0259*	0.062
	174	282	5.38×10^−5^**	0.1354	0.0084*	0.046*
	287	282	5.38×10^−5^**	0.1357	0.0084*	0.046*

a A general linear model not controlling for population structure.

b A mixed linear model controlling for kinship but not population structure.

c A general linear model controlling for population structure (*k* = 5).

d A mixed linear model controlling for both population structure (*k* = 5) and kinship.

Sampling has a larger effect on some sites than others. The 6 bp indel is more significantly associated with flowering time in the smaller population (92 lines) than it is in the 282 association panel analyzed with MLM (K model) without controlling for population structure, but controlling for familial relatedness. The site is, in fact, carried by Northern Flint lines, which are underrepresented in the smaller population. The results for the 282 association panel suggest that the GLM (Q) approach overestimates the association. In contrast, the MLM (Q+K) approach, which accounts for both population structure and relatedness between individuals, gives a moderately significant association between the 6 bp indel (*P*-value = 0.0127) and flowering time variation (Table 1).

The proportion of the genetic variation explained by the different models varies significantly. In this study, the best models are the Q+K and K models (the latter being a MLM that only includes familial relatedness between individuals as random effects) because they explain the highest amount of the genetic variance (Table 2). The reason for the minimal difference between the two models is that K most likely controls for the majority of the relatedness between individuals.

**Table 2:** Genetic variance explained by respective model used for the association study.

Model	SNP	R^2^ Model
Naïve	none	0.000
	6 bp	0.084
	18 bp	0.022
Q	none	0.446
	6 bp	0.461
	18 bp	0.456
K	none	0.898
	6 bp	0.916
	18 bp	0.917
Q+K	none	0.898
	6 bp	0.914
	18 bp	0.914

This study confirms the weak association between the 6 bp indel in *d8* and flowering time analyzed using both GLM and MLM approaches (Table 1). However, the association is not as significant as previously reported by Thornsberry et al. in 2001. Additionally, the GLM and MLM analyses of the 282 association panel imply there is no association between the 18 bp deletion in *d8* and flowering time (Table 1). The initial study by Thornsberry et al. (2001) found this site to be the most significant. Our results from the Q+K and K models yielded a more significant *P*-value for the 92 association panel than the 282 association panel.

We also sequenced a 3 bp indel (266,097,198 bp), which is present in tropical late-flowering lines when we examined sequences available at NCBI. However, new genotypic data for the 282 association panel suggest that there is no association between this site and variation in flowering time in maize (Table 1).

**Figure 1:**
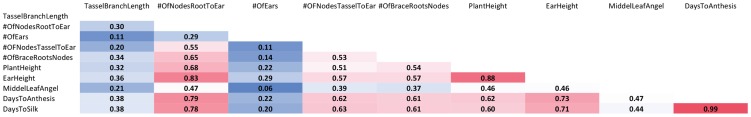
Pearson correlation coefficient between multiple traits. No clear pattern can be observed between correlation between traits and its association to the 6 bp and 18 bp deletions in *d8*.

Our study confirms the results presented by Camus-Kulandaivelu et al. (2008) [37], that there are regions between *d8* and *tb1* associated with variation in flowering time (Table 1) (Figures S3 and S4). However, these sites are moderately significant at α = 0.05 when using the K and Q+K models. Association mapping of *d8* on other traits results in a number of weak associations with other traits, in addition to flowering time (e.g., plant height, ear height, and node number) (Table 3). All the associations are in the same range of significance as flowering time. No clear pattern can be observed between correlation among traits except for what can be expected (e.g., the high correlation between days to silk and days to anthesis) (Figure 1). Collectively, these results undermine the conclusion that *d8* is of more importance for flowering time than any of the other traits.

**Table 3:** Results from association study between polymorphisms within *d8* and a range of traits using MLM (Q+K).

	P-value		
	6 bp	18 bp	3 bp
Days to Silk	0.0013		
Days to Tassel	0.0014		
Number of Ears		0.0469	
Number of Nodes Tassel to Ear		0.0473	0.0083
Middle Leaf Angle	0.0072		
Number of Nodes Ear to Roots	0.0185		
Ear Height	0.0207		
Cob Color	0.0343		
Plant Height	0.0478		
Number of Brace Root Nodes			0.0035
Tassel Branch Length			0.0171

**Figure 2:**
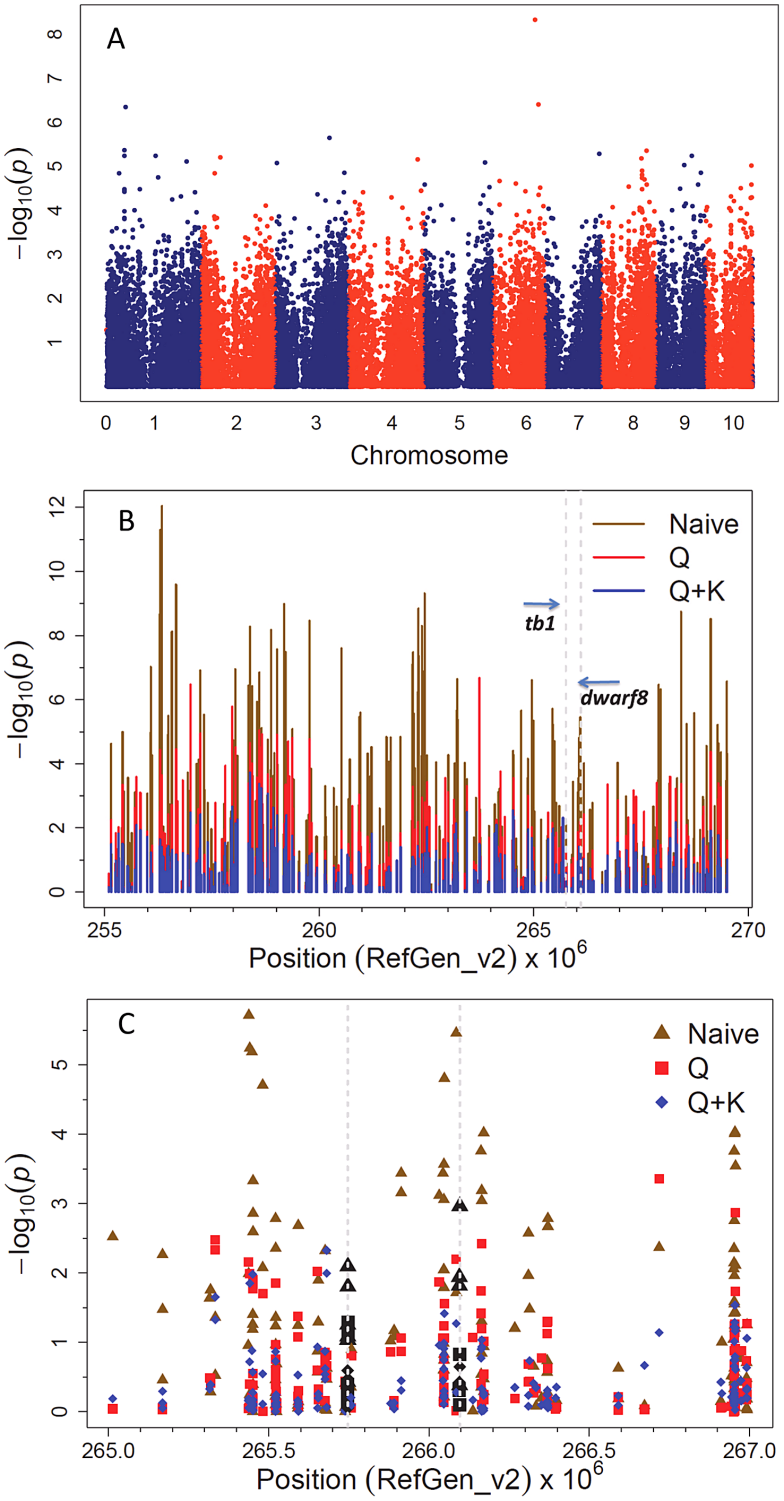
Genome-wide association results for flowering time. A. GWAS results for flowering time (days to silking) in the 282 association panel using genotyping by sequencing (GBS) and 55k SNPs. The Q+K mixed linear model was fitted at each SNP to account for population structure (Q) and kinship (K). Genome-wide association results using the naïve model and Q model in Figures S1 and S2. B. GWAS results for flowering time (days to silking) using three models in the chromosomal region surrounding *tb1* (Chr. 1; 265,745,979–265,747,712 bp) and *d8* (Chr. 1; 266,094,769–266,097,836 bp). All GBS and 55K SNPs between 255 Mb and 270 Mb on Chr. 1 are included in the figure. Brown lines indicate results from naïve model, red lines indicate results from Q model, and blue lines indicate results from Q+K model. C. GWAS results for flowering time (days to silking) using three models in the chromosomal region surrounding *tb1* (Chr. 1; 265,745,979–265,747,712 bp) and *d8* (Chr. 1; 266,094,769–266,097,836 bp). All GBS and 55K SNPs between 265 Mb and 267 Mb on Chr. 1 are included in the figure. Black markers on the right are significant SNPs located within *d8*. Black markers on the left are significant SNPs located within *tb1*. Triangles indicate results from naïve model, squares indicate results from Q model, and diamonds indicate results from Q+K model.

From a genome-wide perspective, there were a large number of sites with a similar degree of association (from the MLM approach) with flowering time as *d8* (Figure 2A). The contrasting results from the various models fitted at the SNPs in the genomic regions surrounding *d8* and *tb1* are illustrated in Figure 2B and 2C. In particular, the GLM model overestimated the significance of the results in comparison to the Q+K and K models. GWAS of flowering time detected SNPs within *d8* that have a weak statistically significant association at α = 0.05 (Figure 2C).

### Linkage Mapping

**Figure 3:**
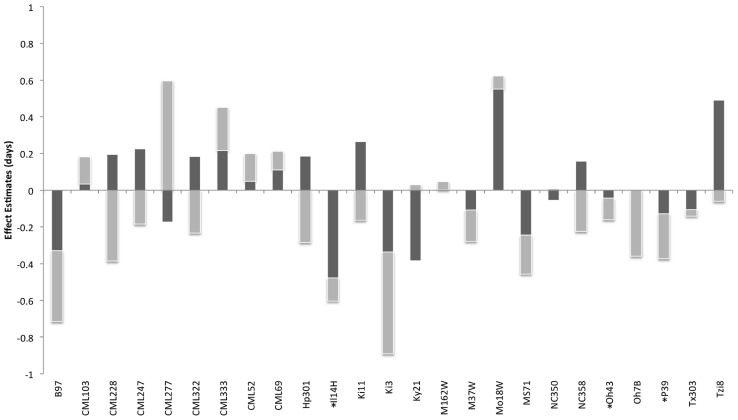
Effect estimates in days for NAM subpopulations carrying QTL in the region of *d8*, *p*-value<0.05. Light gray bar shows QTL effect estimate at marker position 116 (RefGen_v2 position: Chr. 1; 231,701,106–231,703,173 bp) (137.6 cM) on the NAM map. Dark gray bar shows QTL effect estimate at marker position 155 (181.3 cM). *d8* is located closest to marker 135 (RefGen_v2 position: Chr. 1; 269,321,476–269,322,794 bp), (RefGen_v2 position: Chr. 1; 286,977,415–287,063,457 bp), (162.2 cM). * indicates taxa with the 6 bp deletion in *d8*.

Linkage mapping of flowering time in the NAM population detected a number of QTL. A small QTL (*P*-value = 0.0127) colocalized with *d8* (RefGen_v2 position: Chr. 1; 269,321,476–269,322,794 bp), supporting the association identified by association mapping (Figure 3). In the initial study by Thornsberry et al. (2001), the effect of *d8* was estimated to be between 7–10 days. The *d8* polymorphism should be in three of the mapping families, and modest effects are seen in the right direction for all three, but the estimated effect is always less than half a day.

Additionally, many of the subfamilies appear to have other QTL along this section of chromosome 1 (RefGen_v2 position: Chr. 1; 231,701,106–231,703,173 bp and Chr. 1; 286,977,415–287,063,457 bp), but the favored positions are millions of base pairs away. It is quite possible that the mapping position of these joint linkage QTL could be synthetic, but there is little to no support for a QTL in this exact region.

A GWAS in the NAM population for flowering time using 26.5 million segregating SNPs was performed [35], [36]. This approach in the NAM population offers in-depth power and resolution because it utilizes both historic and recent recombination. No significant sites were identified in the region of *d8* (Figure S5). This supports the result that *d8* is not associated with flowering time.

### Haplotype Structure

Hapmap data [35], [36] suggest extended haplotypes for Northern Flint lines in the region of *d8*. Data show modest F_st_ between temperate and tropical subpopulations. However, there could potentially be differences in diversity between these two groups and Northern Flint lines. Hapmap data are only available for a few Northern Flint lines, which limits these studies.

**Figure 4:**
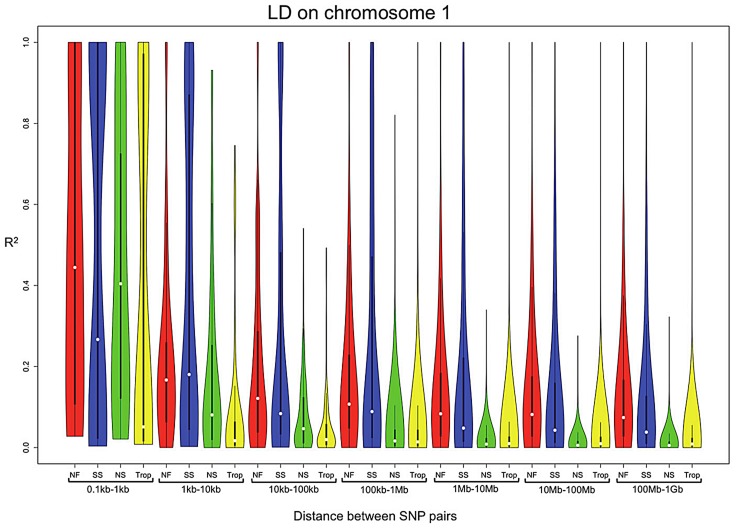
LD on chromosome 1 for the subpopulations, Northern Flint (red), stiff stalk (blue), non-stiff stalk (green), tropical (yellow), of the 282 association panel. White dot indicates median R^2^ for each bin. This graph shows that there is more extended LD in Northern Flint than in other subpopulations.

GBS SNPs were used to examine the range of LD decay within the different subpopulations (Northern Flint, stiff stalk, non-stiff stalk, and tropical) of the 282 association panel. Extended LD is observed for the Northern Flint lines compared to the other subpopulations. Likewise, the stiff stalk lines, which were only founded from 16 inbred lines, also show a pattern of extended haplotypes, although not as extreme as the Northern Flints (Figure 4). The extended haplotype pattern in the Northern Flints make it difficult to control for false positives and to identify the causative SNP using association mapping.

**Figure 5:**
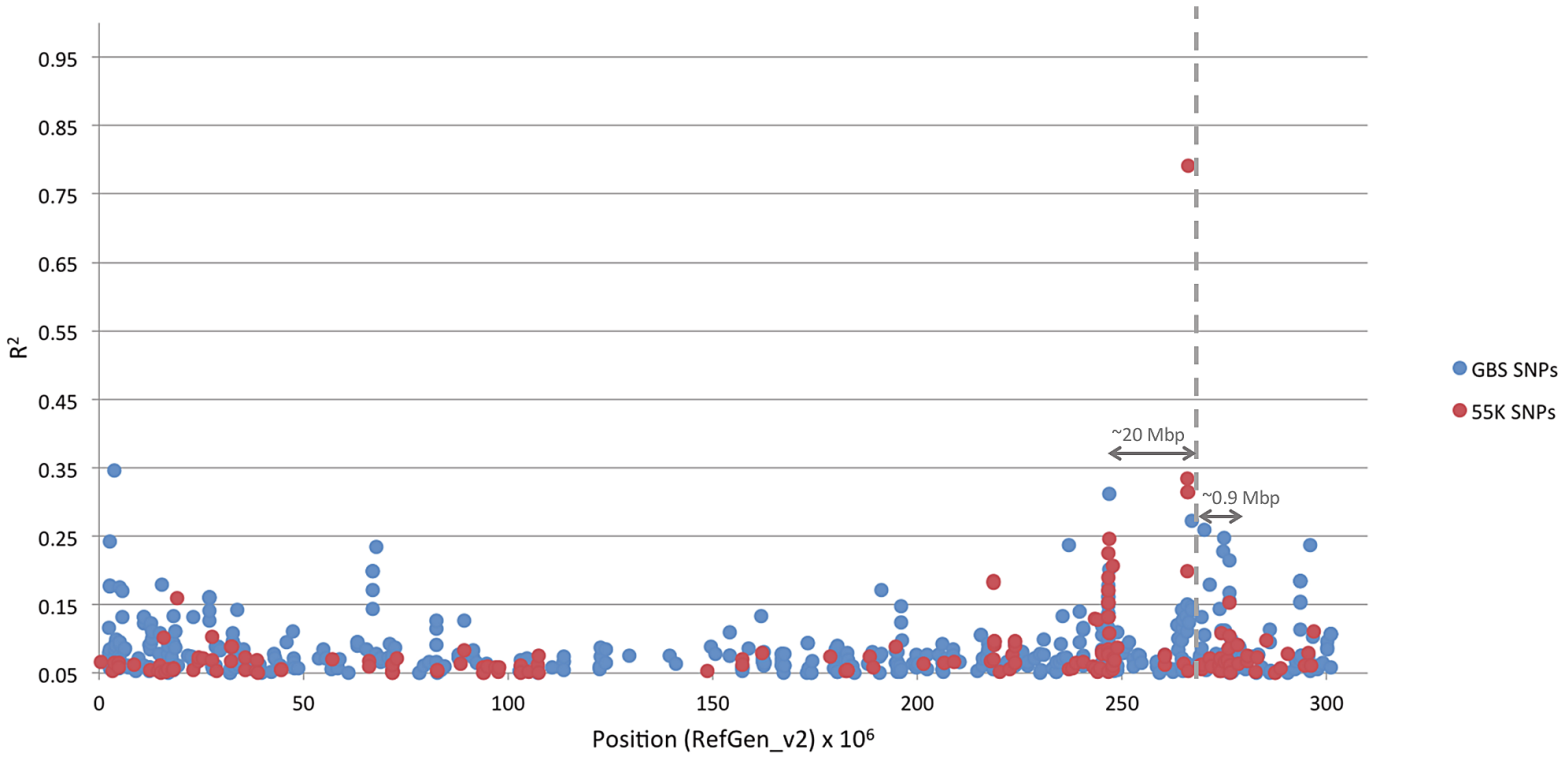
R^2^ between the 6 bp indel in *d8* and all the other sites on chromosome 1. Blue dots indicate results from 13,815 GBS SNPs present in 200 or more of the 282 lines. Red dots indicate results from 7,695 55K SNPs present in 200 or more of the 282 lines.

The 6 bp indel in *d8* is carried by Northern Flint lines. When we examine the LD between the 6 bp indel and the 13,815 high coverage GBS SNPs on chromosome 1 (Figure 5), an extended area around the 6 bp exhibits fairly high values of *R^2^*. This is additional evidence that extended haplotypes exist in the Northern Flint lines in the *d8* region. In fact, there are two regions with high LD at 20 Mbp and 0.9 Mbp away, which contain previously identified domestication gene candidates (i.e., GRMZM2G034217 RefGen_v2 position: chr. 1 246,720,001–247,030,000, a mitochondrial transcription termination factor) [38]. In contrast, LD between the MITE in *vgt1* and the 7,539 GBS SNPs on chromosome 8 (Figure S6) show sites with high *R^2^* values close to the position of the MITE, but LD decays much more rapidly. To test for two-way interaction between the 6 bp and 18 bp indels and the MITE, a series of mixed models including two-way interaction terms were fitted. The most significant interaction was between the 18 bp indel and the MITE (*P*-value 0.0418). However, this association is not likely to be statistically significant after controlling for the multiple testing problem across the entire genome.

## Discussion

### Association Study

The results underscore the importance of properly accounting for population structure in association studies. The analysis in Thornsberry et al. (2001) divided their 92 association panel into three subpopulations. This subdivision did not fully account for population structure, and thus, the effect of the *d8* allele carried by Northern Flint lines was overestimated. In contrast, our study accounted for population structure using both *k* = 3 (stiff stalk, non-stiff stalk, and tropical) and *k* = 5 (stiff stalk, non-stiff stalk, tropical, sweet corn, and popcorn) subpopulations. This was important for sites such as the 6 bp indel within *d8*, which is present at a higher frequency in Northern Flint lines (which includes sweet corn), and this signature of population structure was unaccounted for when *k* = 3 was used.

Of all the models tested, the Q+K model [22] was the most suitable approach for analyzing the 282 association panel because it controls for both population structure and cryptic familial relatedness. It is especially important to control for the latter with traits such as flowering time, which is highly correlated with population structure. Additionally, the Q+K model is beneficial for association studies because Q and K capture different types of long range LD [22], [39].

In general mixed models sufficiently account for population structure and familial relatedness. In contrast, false positives arising from other sources, although rare, are typically unaccounted for in association studies. For example, spurious associations could arise from markers that are in long-range LD with causative polymorphisms. Additionally, causative polymorphisms for one trait may not necessarily be causal for another highly correlated trait (and, hence a spurious association), but will be statistically associated with both traits. Finally, when a trait is controlled by multiple loci in LD, it is likely that the site with the largest effect is an indirect association. One reason for this result arises from differing minor allele frequencies among the causal sites. All three of these types of false positives do not occur randomly across the genome and thus, they are more challenging to eliminate. Haplotype-based association studies is one approach for addressing many of these issues. Nevertheless, multiple sites, selection for multiple traits, and population structure result in spurious associations and these need to be accounted for when performing association studies.

### Contrast with Linkage Mapping

Association mapping is limited when the trait analyzed is correlated with population structure. However, linkage mapping can overcome this problem by crossing individuals with known relatedness, spurious associations can be broken.

In this study, we were able to detect a small QTL at the general location of *d8*. However the favored QTL locations are on both sides of *d8*: RefGen_v2 position Chr. 1; 231,703,173–287,063,457. Association results suggest that the majority of the QTL effects detected around *d8* are from rare extended haplotypes that include other linked QTLs. Another possible explanation for the weakness of the QTLs detected is the population sampled. The associated haplotype is present only in Northern Flint lines, which are underrepresented in the population.

### Haplotype Structure

Flowering time is strongly correlated with population structure. Our study showed that *d8* had a very small effect on flowering time. One possible explanation for this result is that *d8* is associated with another trait that was selected along with flowering time (e.g., cold tolerance). This hypothesis is supported by the extended haplotype pattern observed in Northern Flint lines as well as the associations that are detected with traits like plant height and node number. Northern Flint lines are underrepresented in both the 282 association panel and the NAM population, and it is difficult to scrutinize associations with low allele frequencies. Northern Flint lines have been shown to be distinctive compared to other subpopulations, especially in regions like *d8* and *tb1*, which have been under selection pressure.

Consistent with the findings of previous studies on the *d8* locus, we observed a strong correlation between *d8* and population structure. Thus, the functional site of *d8* is not likely to be involved in flowering time. Indeed, *d8* and *tb1* are strong integrators in plant signals that are adjacent to each other on chromosome 1. However, our results demonstrated that these signals are a signature of population structure instead of true biological signals. The extended LD in the Northern Flint lines around *d8* supports the hypothesis that this gene is regulated in a similar manner as *tb1* and *vgt1*. For both *tb1* and *vgt1*, cis-acting regulatory sites located more than 50 kb from the actual genes have been shown to be the functional regions and not the genes themselves [31], [33], [40].

Signatures of selection on *d8* have been observed in teosinte [41]. Because apical dominance (*tb1*) and gibberellin signaling (*d8*) have both played key roles for domestication phenotypes, it is likely that the genomic region surrounding *d8* and *tb1* has been under selection since early maize domestication. Northern Flint lines differ from Corn Belt dent lines in a number of traits such as leaf angle, plant height, and cold tolerance. Thus, the long range LD block around *d8* could be a signature of selection from the development of Northern Flint lines that happens to be associated with one of these traits distinguishing Northern Flint from Corn Belt dent. Consequently, it has been possible to detect a weak association between flowering time and *d8* because of the correlation between flowering time and the Northern Flint specific traits due to population structure. Using this rationale, it may be possible to detect associations between the *d8* locus and phenotypes such as carbon allocation and harvest index, when considering the differences in the usage of Northern Flints (sweet corn and silage) and Corn Belt dent.

### Conclusion

The basic *d8* associations identified in Thornsberry *et al.* (2001) have been replicated by other independent groups [26], [27], but population structure has always remained a consistent issue. This reanalysis using SNPs within and near *d8* suggests that these associations are either incorrect or vastly overestimated. This work implemented more powerful statistical approaches, germplasm resources, and whole genome sequencing data, enabling a more thorough understanding of this locus.

This analysis underscores the importance of controlling for population structure. All three previously published studies on the *d8* locus illustrate how naïve association results overestimated effect sizes. In our study, we used the unified MLM to control for both population structure and relatedness between individuals, which are more accurate in effect estimation and give a truer level of significance. Even in species with rapid LD decay, like maize, it is possible to have subpopulations that can exhibit LD many orders of magnitude greater than the average length. This long range LD resulted in the extended haplotype lengths observed in Northern Flint lines for the genomic region surrounding *d8*. Northern Flint lines are underrepresented in the association panel, which makes it difficult to accurately account for the population structure of this subpopulation. Another issue is the strong correlations between traits. It is very likely that in the case of *d8* there has been selection for other correlated traits, such as cold tolerance. Because of the correlation between the population structure and flowering time, we can detect a weak association between flowering time and *d8*, but *d8* does not actually have an effect on time of flowering. Genes like *d8* have been targets of strong selection and, as such, are among the hardest to identify in GWAS and accurately estimate their effect size. NAM-like linkage populations with bi-parental crosses in a reference design to minimize population structure may be necessary for dissecting the most structured traits.

Although our results strongly suggest that the previously reported association between *d8* and flowering time is an artifact of population structure, further research on this complex locus is warranted. The long range LD present at *d8* for Northern Flint lines is a signature of selection, and it is important to determine the traits that are regulated by this gene. By applying the appropriate statistical models, we have shown that flowering time is not one of these traits.

## Materials and Methods

### Germplasm

The association panel consists of 282 diverse maize lines that have been previously described [42]. These lines can be subdivided into five major subpopulations, namely stiff stalk, non-stiff stalk, tropical or semitropical lines (related to the non-stiff stalk lines), sweet corn and popcorn. The association panel includes the 25 founder lines of the NAM population. The maize NAM population consists of 5,000 RILs (Recombinant Inbred Lines) derived from crossing B73 with 25 diverse maize inbred lines, and then selfing for 5 generations [43].

### Phenotypic Data

Phenotypic data were collected from the NAM population and the 282 association panel, grown in eight environments including Ithaca, NY, Clayton, NC, Champaign, IL, and Colombia, MO, during the summers of 2006 and 2007. Flowering time was measured separately for female flowers (number of days-to-silk) and male flowers (days-to-anthesis) from the day of planting. The flowering date was defined as the day when the anthers or silk were visible on 50% of all plants within a row.

### Sequencing Data

DNA sequence data were obtained for *d8* from Thornsberry et al. (2001), available at NCBI. Primers were designed for PCR amplification of gene fragments of interest from the 282 lines in the association panel.

Each PCR product was cleaned by treating the samples with Exonuclease (ExoI) and Shrimp Alkaline Phosphatase (SAP) and incubated at 37°C for 3 min followed by 80°C for 10 min. The samples were prepared for sequencing using a mixture with a total volume of 10 µl containing 0.7 µl forward primer, 0.7 µl reverse primer (5 pmol/µl), 0.5 µl Big Dye terminator, 1.7 µl 5× sequencing buffer, 7.1 µl distilled water and the PCR product. The thermal cycler was set on the following program: Initial denaturation at 96°C for 4 min, followed by 30 cycles at 96°C for 10 sec, 50°C for 5 sec and 60°C 4 min, with a final, incubation at 10°C. Sanger(3730XL) DNA sequencing was performed using an Applied Biosystems Automated 3730 DNA Analyzer. The software BioLign alignment and multiple contig editor with codon code phred-phrap analysis was used for alignment using consensus sequence contigs and sequence quality scores.

The alignments from NCBI were also used to reanalyze the results published in the initial study by Thornsberry et al. (2001).

To obtain sequence data for the region between *d8* and *tb1*, the same protocol was used as described above. However, primer sequences were obtained from Camus-Kulandaivelu et al. (2008)

### Statistical Analysis

#### Field spatial correction

Best linear unbiased predictions (BLUPs) of the lines in the 282 association panel and the NAM population were the same as those reported in Buckler et al. (2009). These were obtained from a random effects model fitted in ASREML version 2.0 software [44] that accounts for spatial correlation and field effects.

#### Association mapping—candidate gene study

TASSEL (Trait Analysis by aSSociation, Evaluation, and Linkage) was used for data processing analysis [45], and results were confirmed by using SAS [46]. Association between polymorphisms and phenotypes were evaluated using General Linear Model (GLM) and Mixed Linear Model (MLM) by incorporating phenotypic and genotypic data, population structure (Q) and kinship matrix (K).

Population structure was predicted using a Bayesian approach that estimates the relationship between subpopulations by grouping genotypic correlations at unlinked markers within the population with the software STRUCTURE [18] as described in [42]. This approach uses the proportion of an individual's genome that originated from each subpopulation to estimate the genetic background matrix (Q).

In MLM, the familial relatedness between the individuals is taken into consideration through a kinship matrix. This model corrects for spurious associations arising from population structure and familial relatedness [47]. In this study we used marker-based kinship, which was determined on the basis of the definition that random pairs of inbreds are unrelated. Kinship was calculated using the software package SPAGeDi [48]. It has been suggested that marker based kinship is more appropriate for association studies than kinship based on pedigree records [19], [40]. The same set of markers was used to create the population structure and kinship matrix.

The GLM approach in this study for phenotype, **y**, is:
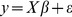
(1)Where, the **Xβ** term represents the fixed effects, including genotypes and population structure, Q, and **ε** is a vector of residual effects following a multivariate normal distribution with mean 0 and variance-covariance matrix σ^2^_ε_I. The naïve model is the same as GLM without the population structure effect.

The MLM approach in this study is the same model as used by Yu et al., 2006.

Mixed model, for phenotype, **y**, is:
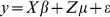
(2)Where, the **Xβ** term represents the fixed effects, including genotypes and population structure, Q, and the **Zμ** term represents random line effects, including the matrix of kinship coefficients, K, and vector of polygene background effects. **ε** is a vector of residual effects following a multivariate normal distribution with mean 0 and variance-covariance matrix σ^2^_ε_I.

#### Genome-wide association study

Genome-wide association studies (GWAS) were carried out in the 282 association panel using 51,741 SNPs obtained from the Illumina MaizeSNP50 BeadChip and 591,552 SNPs from the genotyping by sequencing (GBS) protocol [49]. Three different approaches that take into account varying degrees of population structure and familial relatedness were undertaken. The first approach, called the naïve approach, uses a model similar to the one presented in Equation (1), except that the Q matrix representing population structure is not included among the fixed effects. The next approach is the GLM approach, which uses the model in Equation (1), with the first five principal components (PCs) of the non-industry subset of the Illumina MaizeSNP50 BeadChip SNPs (34,368 SNPs) included as fixed effects to represent population structure. The final approach is the MLM approach, with the aforementioned first five PCs representing population structure, and a kinship matrix calculated from the non-industry subset of these SNPs for the variance-covariance matrix of the random line effects. This kinship matrix is calculated using the method of [50]. In each approach, these models are fitted to each SNP. After all SNPs with minor allele frequencies (MAFs) less than 0.05 are removed from the analysis, the Benjamini-Hochberg [51] procedure adjusts for the multiple testing problem by controlling the false discovery rate (FDR) at 0.05. This phase of the anlaysis was conducted using the genome association and prediction integrated tool (GAPIT) package in the R programming language [52].

#### Joint-Linkage Mapping

Joint Linkage Mapping of BLUPs for the phenotype across environments was performed using the proc GLMSelect in SAS, as described in Buckler et al. (2009) [11]. BLUPs were calculated for each phenotype and used together with imputed genetic marker intervals and stepwise regression to identify QTLs. Missing marker data were imputed by utilizing genetic distance between missing and flanking markers. A permutation procedure was implemented to obtain empirical α = 0.05 thresholds for including and excluding terms in the joint linkage model [53].

#### Linkage Disequilibrium

To calculate the linkage disequilibrium between the SNPs within *d8*, tested in this association study, against the rest of the genome the LD function SitebyAll in the TASSEL software was used [45]. For genotypic data, the Illumina MaizeSNP50 Beadchip was used, as well as 458k GBS (Genotyping by Sequencing) SNPs [49].

## Supporting Information

Figure S1 Genome-wide association results for flowering time (days to silking) in the 282 association panel using genotyping by sequencing (GBS) and 55k SNPs. The naïve model, which does not account for population structure, was fitted at each SNP.
(TIF)

Figure S2 Genome-wide association results for flowering time (days to silking) in the 282 association panel using genotyping by sequencing (GBS) and 55k SNPs. The Q model was fitted at each SNP to account for population structure (Q).
(TIF)

Figure S3 Physical positions of *tb1* and *d8* on RefGen_v2. Positions of SNPs are obtained by blasting primer sequences using www.maizesequence.org and are approximate. Sites above the line in solid black boxes are evaluated in this study. Sites below the line in dashed boxes are from the study by Camus-Kulandaivelu et al. (2008).
(TIF)

Figure S4 The region around *tb1* and *d8* on chromosome 1 (265,495,979–266,347,836 RefGen_v2), and all identified gene transcripts available at www.maizesequence.org. There are no other obvious candidate genes for flowering time in the region.
(TIF)

Figure S5 Genome-wide association results for flowering time (days to silking) in the NAM population using maize HapMapv1 and HapMapv2 SNPs. There are no significant sites identified in the region of *d8* (indicated by the gray line).
(TIF)

Figure S6 R^2^ between MITE in *vgt1* and all the other sites on chromosome 8. Blue dots indicate results from 7,539 GBS SNPs present in 200 or more of the 282 lines. Red dots indicate results from 4,197 55K SNPs present in 200 or more of the 282 lines.
(TIF)
