# Supplementary figures and images for: Lessons from Dwarf8 on the Strengths and Weaknesses of Structured Association Mapping
Source: PLoS Genet. 2013 Feb 21;9(2):e1003246. doi: 10.1371/journal.pgen.1003246 (PMC3578782; doi:10.1371/journal.pgen.1003246)

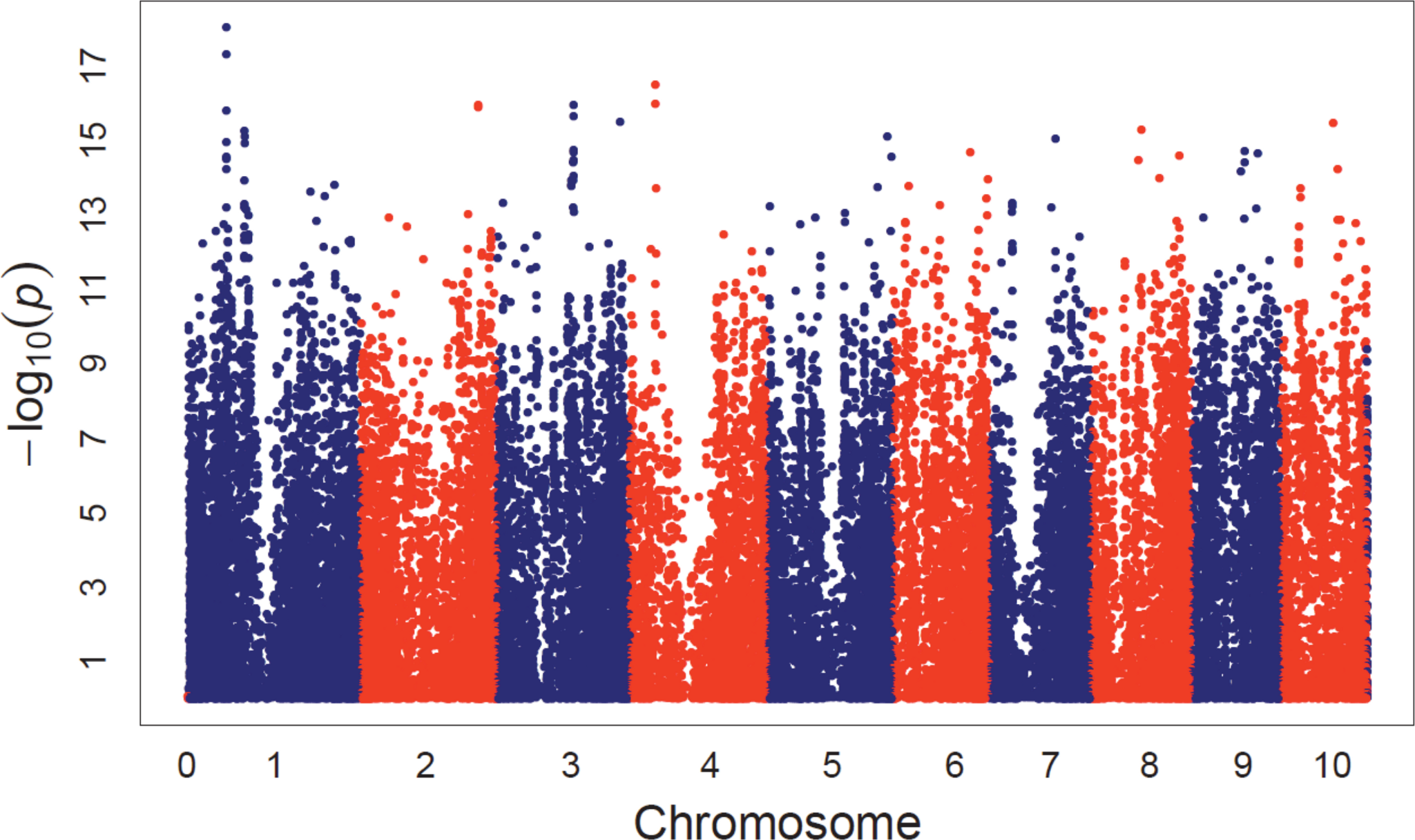

Supplement: Figure S1 — Genome-wide association results for flowering time (days to silking) in the 282 association panel using genotyping by sequencing (GBS) and 55k SNPs. The naïve model, which does not account for population structure, was fitted at each SNP. (TIF) [file pgen.1003246.s001.tif]

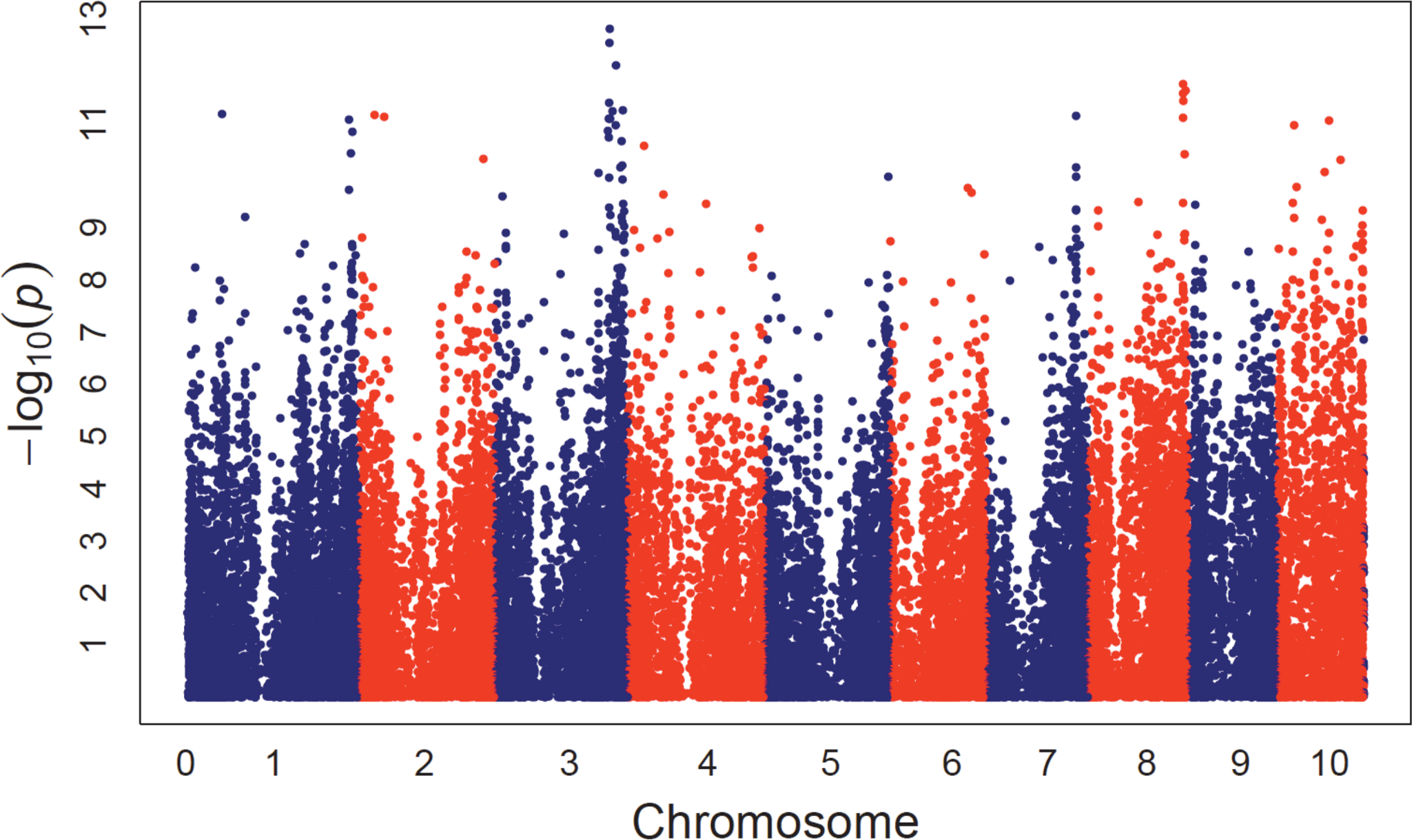

Supplement: Figure S2 — Genome-wide association results for flowering time (days to silking) in the 282 association panel using genotyping by sequencing (GBS) and 55k SNPs. The Q model was fitted at each SNP to account for population structure (Q). (TIF) [file pgen.1003246.s002.tif]

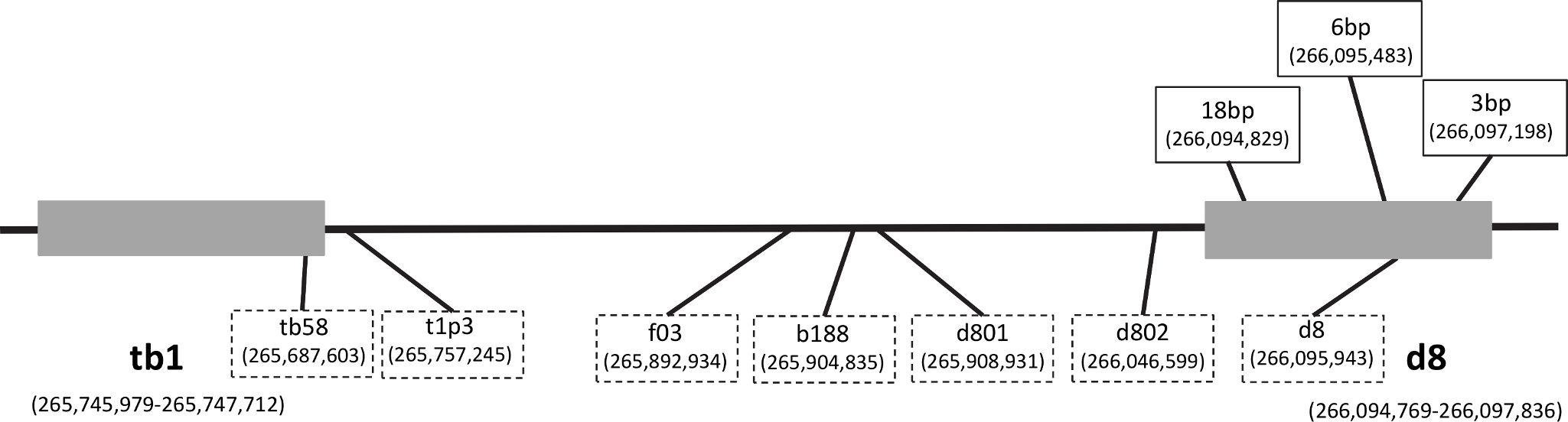

Supplement: Figure S3 — Physical positions of tb1 and d8 on RefGen_v2. Positions of SNPs are obtained by blasting primer sequences using www.maizesequence.org and are approximate. Sites above the line in solid black boxes are evaluated in this study. Sites below the line in dashed boxes are from the study by Camus-Kulandaivelu et al. (2008). (TIF) [file pgen.1003246.s003.tif]

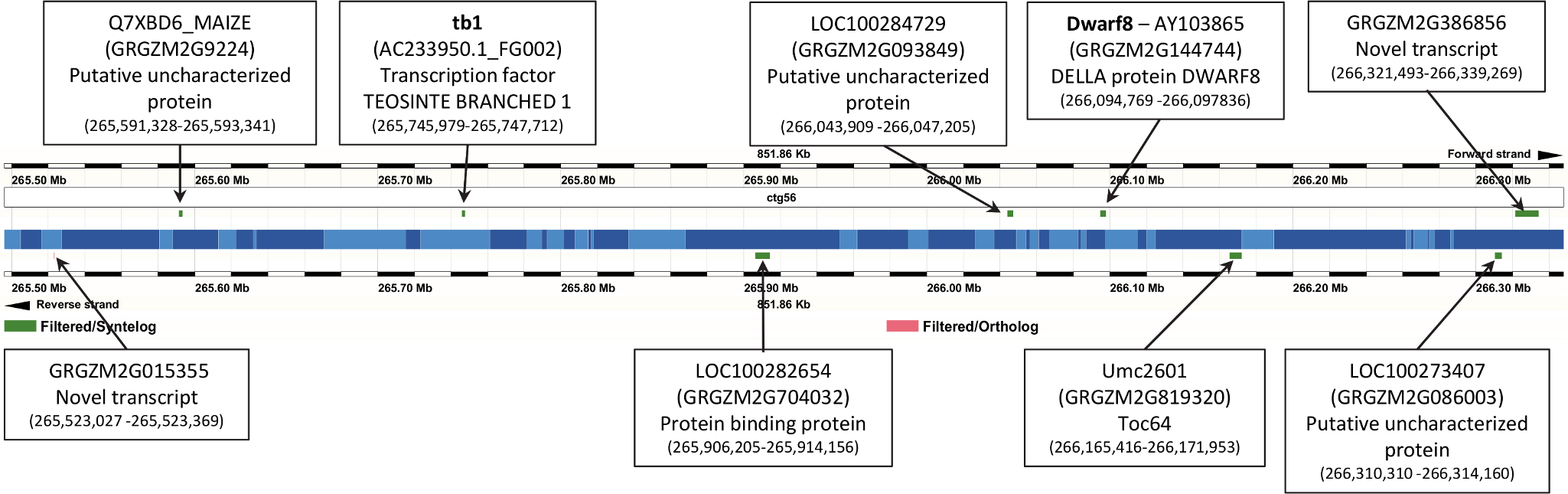

Supplement: Figure S4 — The region around tb1 and d8 on chromosome 1 (265,495,979–266,347,836 RefGen_v2), and all identified gene transcripts available at www.maizesequence.org. There are no other obvious candidate genes for flowering time in the region. (TIF) [file pgen.1003246.s004.tif]

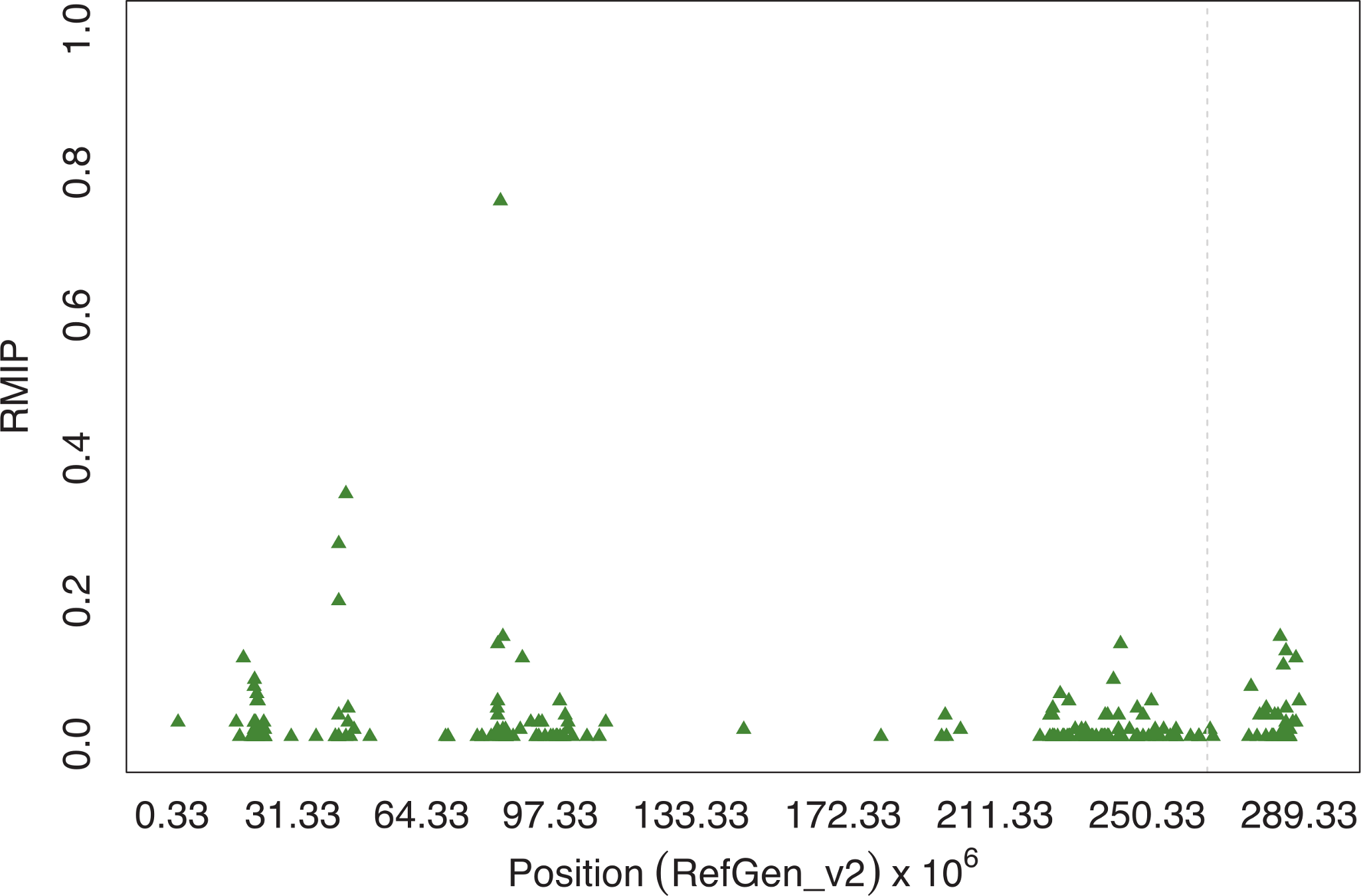

Supplement: Figure S5 — Genome-wide association results for flowering time (days to silking) in the NAM population using maize HapMapv1 and HapMapv2 SNPs. There are no significant sites identified in the region of d8 (indicated by the gray line). (TIF) [file pgen.1003246.s005.tif]

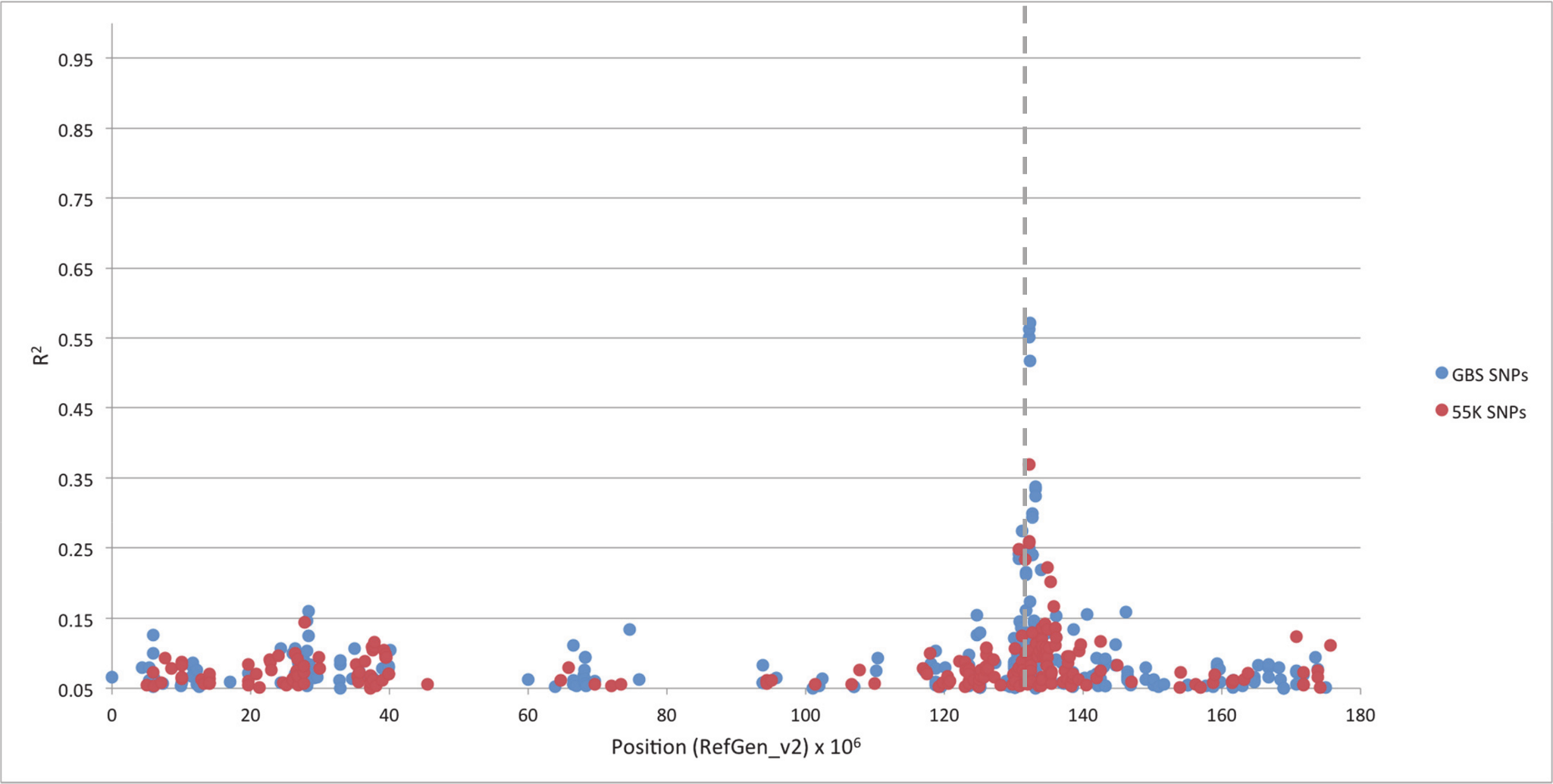

Supplement: Figure S6 — R2 between MITE in vgt1 and all the other sites on chromosome 8. Blue dots indicate results from 7,539 GBS SNPs present in 200 or more of the 282 lines. Red dots indicate results from 4,197 55K SNPs present in 200 or more of the 282 lines. (TIF) [file pgen.1003246.s006.tif]
